# Increased Expression of *LASI* lncRNA Regulates the Cigarette Smoke and COPD Associated Airway Inflammation and Mucous Cell Hyperplasia

**DOI:** 10.3389/fimmu.2022.803362

**Published:** 2022-06-14

**Authors:** Marko Manevski, Dinesh Devadoss, Christopher Long, Shashi P. Singh, Mohd Wasim Nasser, Glen M. Borchert, Madhavan N. Nair, Irfan Rahman, Mohan Sopori, Hitendra S. Chand

**Affiliations:** ^1^ Department of Immunology and Nano-Medicine, Herbert Wertheim College of Medicine, Florida International University, Miami, FL, United States; ^2^ Respiratory Immunology Program, Lovelace Respiratory Research Institute, Albuquerque, NM, United States; ^3^ Department of Biochemistry and Molecular Biology, University of Nebraska Medical Center, Omaha, NE, United States; ^4^ Department of Pharmacology, University of South Alabama, Mobile, AL, United States; ^5^ Department of Environmental Medicine, University of Rochester Medical Center, Rochester, NY, United States

**Keywords:** bronchial epithelial cells, cigarette smoke (CS), chronic obstructive pulmonary disease (COPD), long noncoding RNA (lncRNA), mucus hyperexpression, lncRNA antisense to ICAM-1 (LASI)

## Abstract

**Research Impact:**

Cigarette smoke (CS) exposure is strongly associated with chronic obstructive pulmonary disease (COPD). In respiratory airways, CS exposure disrupts airway barrier functions, mucous/phlegm production, and basic immune responses of airway epithelial cells. Based on our recent identification of a specific immunomodulatory long noncoding RNA (lncRNA), we investigated its role in CS-induced responses in bronchial airways of cynomolgus macaque model of CS-induced COPD and in former smokers with and without COPD. The lncRNA was significantly upregulated in CS-induced macaque airways and in COPD airways that exhibited higher mucus expression and goblet cell hyperplasia. Experimental models of cells derived from COPD subjects recapitulated the augmented inflammation and mucus expression following the smoke challenge. Blocking of lncRNA expression in cell culture setting suppressed the smoke-induced and COPD-associated dysregulated mucoinflammatory response suggesting that this airway specific immunomodulatory lncRNA may represent a novel target to mitigate the smoke-mediated inflammation and mucus hyperexpression.

**Rationale:**

In conducting airways, CS disrupts airway epithelial functions, mucociliary clearances, and innate immune responses that are primarily orchestrated by human bronchial epithelial cells (HBECs). Mucus hypersecretion and dysregulated immune response are the hallmarks of chronic bronchitis (CB) that is often exacerbated by CS. Notably, we recently identified a long noncoding RNA (lncRNA) antisense to ICAM-1 (*LASI*) that mediates airway epithelial responses.

**Objective:**

To investigate the role of *LASI* lncRNA in CS-induced airway inflammation and mucin hyperexpression in an animal model of COPD, and in HBECs and lung tissues from former smokers with and without COPD. To interrogate *LASI* lncRNA role in CS-mediated airway mucoinflammatory responses by targeted gene editing.

**Methods:**

Small airway tissue sections from cynomolgus macaques exposed to long-term mainstream CS, and those from former smokers with and without COPD were analyzed. The structured-illumination imaging, RNA fluorescence *in-situ* hybridization (FISH), and qRT-PCR were used to characterize lncRNA expression and the expression of inflammatory factors and airway mucins in a cell culture model of CS extract (CSE) exposure using HBECs from COPD (CHBEs) in comparison with cells from normal control (NHBEs) subjects. The protein levels of mucin MUC5AC, and inflammatory factors ICAM-1, and IL-6 were determined using specific ELISAs. RNA silencing was used to block *LASI* lncRNA expression and lentivirus encoding *LASI* lncRNA was used to achieve *LASI* overexpression (LASI-OE).

**Results:**

Compared to controls, *LASI* lncRNA was upregulated in CS-exposed macaques and in COPD smoker airways, correlating with mucus hyperexpression and mucus cell hyperplasia in severe COPD airways. At baseline, the unstimulated CHBEs showed increased *LASI* lncRNA expression with higher expression of secretory mucin MUC5AC, and inflammatory factors, ICAM-1, and IL-6 compared to NHBEs. CSE exposure of CHBEs resulted in augmented inflammation and mucus expression compared to controls. While RNA silencing-mediated *LASI* knockdown suppressed the mucoinflammatory response, cells overexpressing *LASI* lncRNA showed elevated mRNA levels of inflammatory factors.

**Conclusions:**

Altogether, *LASI* lncRNA may represent a novel target to control the smoke-mediated dysregulation in airway responses and COPD exacerbations.

## Introduction

COPD is the third leading cause of death globally, with over 300 million diagnosed cases and over 3 million COPD-related fatalities annually (1, 2). Environmental exposure to toxins or allergens exacerbates the disease and are associated with a very high (55%) 5-year mortality rate among COPD patients (3). COPD is an aging-associated condition characterized by progressive and irreversible airway obstruction and tissue remodeling which presents as chronic bronchitis (CB) and alveolar tissue destruction (4). CB patients often suffer from dyspnea, cough and sputum production leading to increased rates of exacerbation, accelerated lung tissue aging and decline in lung function, reduced quality of life, and increased mortality (4, 5). CB is more prevalent among COPD patients, with approximately 74% patients reportedly affected with CB (5).

Among the environmental factors, cigarette smoke (CS) is the primary risk factor associated with COPD development. CS exposure induces inflammatory pathways and epithelial tissue remodeling in conducting airways through goblet cell hyperplasia and the loss of ciliary cells (2). CS exposure induces epigenetic events such as DNA methylation, histone modifications and notably, changes in expression of noncoding transcripts such as microRNAs (miRNAs) and long noncoding RNAs (lncRNAs) (6, 7). LncRNAs are noncoding transcripts over 200 bases in length and may interact with proteins, DNA, chromatin, and other RNAs to induce epigenetic and transcriptomic changes, affecting cell and tissue functions (8). A growing number of lncRNAs have been implicated in chronic pulmonary conditions, including CS-related immune responses, and inflammatory dysregulation (6, 9, 10). LncRNA-mediated regulation of immune responses is potentially central to the establishment of innate mucosal immunity, and dysregulated expressions may promote hyperactive immune responses like those observed in COPD subjects, following exacerbations. To understand the molecular mechanisms through which lncRNA regulate innate immune responses, we recently identified novel lncRNAs that impact lung pathology. Specifically, we identified a specific immunomodulatory lncRNA referred to as *LASI*, a lncRNA antisense to ICAM-1, and confirmed its direct involvement in modulating airway inflammatory and mucus hyperexpression responses (11).

We recently observed that long-term mainstream CS exposure results in airway remodeling in cynomologus macaques (*Macaca fascicularis*) with augmented chronic bronchitis (CB) and reduction in lung functions similar to those observed in smoke-associated COPD (12). Based on the important role of lncRNAs in COPD (6) and specifically *LASI* lncRNA in airway epithelial cells (11), we hypothesized that *LASI* lncRNA affected the CS-induced airway inflammation and mucus hypersecretion in COPD. Herein, using the structured-illumination imaging and RNA FISH, we found that *LASI* lncRNA was upregulated in the small airway epithelium of an animal model of chronic CS exposure as well as in former smokers with COPD, and its expression correlates with aggravated inflammatory and mucus secretory responses. In addition, unstimulated airway cells from COPD subjects showed strong association of higher *LASI* expression with upregulated expression of mucin and other inflammatory factors. Most importantly, we find blocking *LASI* expression rescues impaired airway responses due to CS-mediated dysregulations in COPD bronchial epithelial cells and the ectopic overexpression of LASI lncRNA resulted in increased expression of airway epithelial inflammatory factors.

## Materials and Methods

### Human Lung Tissue Samples

Lung tissue samples were obtained from the Lung Tissue Research Consortium (LTRC) from the National Institutes of Health (NIH). The COPD patient cohorts are defined as patients with a post-bronchodilator FEV1/FVC <0.7, currently the most widely accepted and robust test for COPD. Although reports have shown that early-stage COPD may present with emphysematous or other pathologic changes prior to a presentation of an FEV1/FVC <0.7, this test remains the standard confirmation of COPD diagnosis. COPD patient samples were compared to samples from GOLD stage 0 patients, which are defined as having normal spirometry results, however, may have chronic symptoms such as cough and sputum product, and may present with risk factors for COPD such as smoking behavior (13). GOLD 0 individuals may or may not progress to active COPD status and are classified as pre-COPD (14). Both male and female subjects in each GOLD stage were grouped together and all groups include both active and former smokers. GOLD stage determination was made by spirometry testing and assigned to the appropriate GOLD stage group, per the protocols described by the National Heart, Lung, and Blood Institute and World Health Organization (13). In this report, GOLD stages I and II were defined as patients with mild COPD status and GOLD stages III and IV were defined as patients with severe COPD status. Each group had a minimum n=6 with a mean age between 59.7 and 65.2 years old. All COPD patients had a smoking history with average packs per year (PY) ranging between 22.4 and 41.5 and former smokers had not been smoking for an average of 13.2 and 22.9 years. Smoking history was self-reported for all patients. Lung tissue homogenates include epithelial tissue as well as other tissues and cell types. Samples were obtained from varying anatomical regions of the lungs, however all samples contained bronchial epithelial cells as confirmed by expression of pan-cytokeratin (pan-CK) from epithelial cells and MUC5AC mucin from secretory goblet cells.

### *M. fascicularis* Cigarette Smoke Exposure

Female cynomolgus macaques (*M. fascicularis*) were exposed to mainstream CS in H2000 whole body exposure chambers at 250 mg/m^3^ total suspended particulate matter (TPM) for 6 hours per day, 5 days per week, corresponding to 4 packs of cigarettes per day as described previously (12). At these concentrations C. macaques develop observable changes in lung physiology within three months of exposure. Lung resections and tissue sections were obtained as described previously (12).

### Human Bronchial Epithelial Cell Culture and CSE Treatment

The primary HBECs from COPD and non-COPD donors were obtained from the commercial suppliers (Lonza Inc. or MatTek Corp). All primary cell lines were grown and treated in bronchial epithelial cell growth media (BEGM from Lonza or UNC MLI Tissue Procurement and Cell Culture Core). Air-liquid interface (ALI) cultures of primary cells were grown in BEGM and differentiated in bronchial ALI (B-ALI) differentiation media (Lonza or UNC MLI Tissue Procurement and Cell Culture Core), as described previously (11). ALI cultures were seeded at a density of 5 x 10^5^ cells/cm^2^ on collagen IV-coated Costar^®^ 6.5 mm Transwells with 0.4 µm pore polyester membranes (Corning Costar Corporation). Cells were differentiated for a minimum of 21 days before treatments. Epithelial differentiation was confirmed by live cell imaging of ciliary beatings and mucus glycoprotein expression. For CSE preparation, CS particulate matter collected on the filter membranes from mainstream smoke of 3RF4 research cigarettes (courtesy Philip Kuehl, Lovelace Biomedical) were used and final treatments at 20 µg/ml CSE were used. Apical side epithelial cells were exposed to treatments for 30 minutes at each treatment point. In addition, paraffin-embedded tissue sections human COPD and healthy control lungs were obtained from the LTRC.

### Quantitative Real-Time PCR With Reverse Transcription (qRT-PCR)

For all RT-qPCR analysis, total RNA extraction was performed using the RNeasy Mini kit (Qiagen) according to manufacturer’s instructions. RNA concentration was quantified using the Synergy HTX reader (BioTek, VT). Complementary (c)DNA was synthesized using the iScript Advanced cDNA synthesis kit (Bio-Rad), per manufacturer’s instructions. FAM-based and SYBR Green primers were used. The *LASI, ICR, WAKMAR-2, NEAT1, MALAT1* lncRNAs and *MUC5AC* and *SPDEF* mRNA levels were quantified using FAM-based primer/probe sets and TaqMan gene expression kit or the SsoFast qPCR master mix (Applied Biosystems, Thermo Fisher). *ICAM-1*, *IL6*, and *CXCL8* mRNA levels were quantified using SYBR Green-based primers and the iTaq master mix (Bio-Rad). qRT-PCR was conducted using the Bio-Rad CFX Real-Time PCR detection system. Quantification of the results was performed using the delta-delta (ΔΔ)Ct method and U6 noncoding small nuclear RNA (snRNA) was used as a reference gene for lncRNA expression levels and *beta-actin* and *GAPDH* were used for mRNA expression levels as described recently (15).

### RNA Fluorescence *In-Situ* Hybridization (FISH)

The RNAScope^®^ 2.5 HD duplex assay and reagent kit (Advanced Cell Diagnostics, Biotechne) was used for RNA FISH as per the manufacturer’s instructions. A double-Z probe set against *LASI* was designed containing 20 dual probes targeting various segments across the *LASI* lncRNA. RNA FISH was conducted on paraffin-embedded 5 µm tissue sections obtained from the LTRC of the NIH. Deparaffinization was conducted in consecutive xylene, graded ethanol, and deionized water. Pretreatment was conducted with hydrogen peroxide solution and the RNAScope^®^ target retrieval buffer and protease plus solutions were used to exposure the antigen. Probe hybridization was conducted for 2 hours at 40°C in the HybEZ^®^ II oven. The signal was amplified using the Amp1, Amp2, Amp3 and the HRP probe at 40°C in the HybEZ^®^ II oven. The signal was detected using the tyramide signal amplification (TSA) reaction with an Alexa fluor labeled TSA kit (Perkin Elmer). Sections were then processed for immunohistochemistry or directly mounted with the 4’,6-diamidino-2-phenylindole (DAPI)-containing Fluormount-G (Southern Biotechnology). Images were captured using the Keyence BZ-X700 structured illumination fluorescent microscope. Analysis was conducted with the Keyence BZ-X analysis software and using the ImageJ software (NIH). RNA FISH quantification was conducted according the RNAScope^®^ histo (H)-score methodology. In each image, probe signals were counted for each cell, both in the nuclear and cytosolic region and assigned to appropriate bins: bin 0 (no signals), bin 1 (1-3 signals/cell), bin 2 (4-9 signals/cell), bin 3 (10-15 signals/cell) and bin 4 (>15 signals/cell). The H-score was calculated as follows: H-score was the sum of each bin multiplied by the percentage of cells that fall into that bin. H-score = (0 x % cells in bin 0) + (1 x % cells in bin 1) + (2 x % cells in bin 2) + (3 x % cells in bin 3) + (4 x % cells in bin 4). Final H-scores ranged from 0 to 400 per group.

### Immunohistochemistry

Tissue sections or fixed cells were washed in 0.05% V Brij-35 in PBS+. Antigen retrieval was performed using 10 mM citrate buffer (pH 6.0). Blocking solution (1% NDS, 3% BSA, 1% gel, 0.2% TX-1000 and 0.2% saponin in PBS+) incubation was conducted for 1 hour at room temperature followed by incubation at 4°C overnight with primary antibodies against mucin MUC5AC (Millipore Sigma) and pan-cytokeratin (pan-CK, Santa Cruz Biotechnology). Appropriate DyLight^®^ fluorescently-conjugated secondary antibodies (Abcam) were used, and sections were incubated for 1 hour at room temperature. Sections were mounted with DAPI-containing Fluormount-G. Immunofluorescent images were captured using the Keyence BZ-X700 microscope and image analysis was conducted using the ImageJ software (NIH). Mean fluorescence intensity per number of epithelial cells was used to compare mucin MUC5AC expression levels. Pan-CK was used as a confirmation of epithelial cell identity.

### Histochemical Analyses

Tissue sections were deparaffinized and hydrated in graded ethanol and deionized water. Histochemical staining was conducted with Alcian blue-period acid Schiff (AB-PAS) or AB followed by hematoxylin and eosin (H&E) or AB-H&E staining as described (16). The mucus secretory cells (goblet/mucous cells) were quantified as a total number of AB-PAS+ or just AB+ cells per mm basal lamina for each image.

### Enzyme-Linked Immunosorbent Assays (ELISAs)

Culture media and apical cell culture washes from NHBEs and CHBEs differentiated in 3D ALI culture for 28 days was collected prior to CSE treatment and every two days after treatment. Final apical washes and culture media supernatant were collected prior to the termination of the experiment and was either stored at -80°CC or processed for analysis. The protein levels of MUC5AC, ICAM-1, and IL-6 were determined using human ELISA kits against MUC5AC (MyBioSource Inc., San Diego, CA), ICAM-1 (LifeSpan Biosciences Inc., Seattle, WA), and IL-6 (BioLegend Inc., San Diego, CA), respectively, as per manufacturers’ instructions.

### Immunofluorescent Cytometry

For immunostaining analyses, cell cultures grown in Nunc™ Lab-Tek™ II 8-chamber slide system were washed using 0.05% v Brij-35 in PBS(+) and immunostained as described previously (11). Cells were stained with antibodies to mucin MUC5AC (Millipore, Inc.), ICAM-1 (Cell Signaling Technology, Inc.) and pan cytokeratin (Cell Signaling Technology, Inc.). Immunostained cells were detected using respective secondary fluor-conjugated antibodies (Thermo Fisher Scientific, Inc) and mounted with DAPI-containing mounting media. Immunofluorescent images were captured using a Keyence BZ-X710 all-in-one fluorescence microscopy system and were analyzed using Keyence analysis software and Image J software (National Institutes of Health). Labtech slides of NHBEs and CHBEs treated with or without CSE were immunostained for MUC5AC or ICAM-1 separately. Labtech slides stained for either MUC5AC or ICAM-1 were imaged, three random images per well were captured with 2 wells per treatment and a total of 200 cells were counted per treatment group and the percent positive cells were calculated, as described earlier (11).

### lncRNA *LASI* Overexpression

A full-length *LASI* lncRNA sequence was cloned into a pLenti-GIII-CMV-GFP-2A-Puro vector and a high titer lentivirus preparation was obtained from Applied Biological Materials Inc. (Richmond, Canada). Primary HBECs cultured overnight in 6-well plates were transduced with *LASI-*overexpression (LASI-OE) lentiviral preparation at 0.5 and 2 MOI (multiplicity of infection) using a culture media containing 8 µg/ml of polybrene (Sigma). Set of control cells were also transduced similarly with empty vector lentiviral preparation (Lenti-EV) at 0.5 and 2 MOI. The GFP-tag fluorescence was followed to assess the transduction efficiency. Forty-eight-hour post-transfection, cells were harvested, and qPCR was performed for expression analysis of *LASI* lncRNA and other transcripts. Mock-transduced cells (or cells with 0 MOI) were used as controls to analyze the expression levels.

### Statistical Analysis

Grouped results were expressed as mean ± SEM and *p*<0.05 was considered significant. Data were analyzed using GraphPad Prism Software (GraphPad Software Inc.) using one-way analysis of variance (ANOVA) with and Tukey’s multiple comparison test or using a two-tailed t test for comparison between two groups. When significant main effects were detected (*p*<0.05), student’s t test was used to determine differences between the groups. All *in-vitro* studies were performed following 3 separate experiments. NHBEs and CHBEs were obtained from three different donors for the baseline and the CSE-treatment analysis.

## Results

### Chronic CS Exposure Results in Goblet/Mucous Cell Hyperplasia and Increased *LASI* lncRNA Expression in Bronchial Airway Epithelium

**Figure 1:**
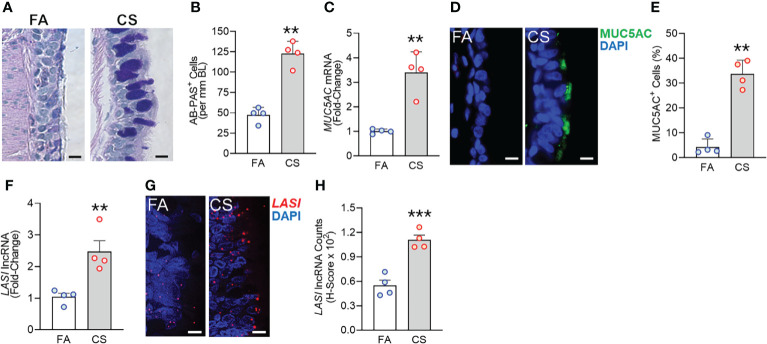
Chronic cigarette smoke (CS) exposure of cynomolgus macaques results in goblet/mucous cell hyperplasia that is strongly associated with increased *LASI* lncRNA expression in bronchial airways. Bronchial airway tissues from C. macaques exposed to mainstream CS or control filtered-air (FA) for 27 weeks as reported recently (12) were analyzed. **(A)** Representative histomicrographs of bronchial tissue sections showing AB-PAS-stained goblet/mucous cells in CS- and FA-exposed macaques, scale – 10 µm. **(B)** Number of AB-PAS+ cells quantified per mm of basal lamina (BL) in each group. **(C)** Relative quantity of secretory mucin *MUC5AC* mRNA in CS-exposed macaques compared to FA-controls as determined by qPCR. **(D)** Representative micrographs of bronchial tissue sections showing MUC5AC immunopositivity in CS- and FA-exposed macaques, scale – 5 µm. **(E)** Percentage of MUC5AC+ cells over the total epithelial cells quantified for each group. **(F)** Relative quantity of *LASI* lncRNA in CS-exposed macaques versus FA-controls as determined by qPCR. **(G)** Representative micrographs showing *LASI* lncRNA expression in FA and CS macaques as determined by FISH, scale – 5 µm. **(H)** Quantification of LASI lncRNA expression in bronchial epithelial cells of each group as determined by H-score analysis described earlier (11). Data shown as mean ± SEM; n=4/gp and analyzed by student’s t-test; **p<0.01; ***p<0.001.

We recently analyzed the effects of long-term CS exposure in a large animal model where cynomologus macaques (*M. fascicularis*) were exposed to mainstream CS for twenty-seven weeks, where CS-exposed macaques showed COPD-like respiratory phenotypes (12). Here, we specifically analyzed the bronchial epithelial responses in archived lung tissues of CS-exposed macaques and filtered room-air (FA) exposed control macaques (n=4 each). Compared to FA group, CS-exposed macaque lungs showed significant bronchial airway epithelial remodeling with augmented goblet/mucous cell hyperplasia (**Figure 1A**) as analyzed by histochemical staining using AB-PAS reagent. There were 2.6-fold higher number of AB-PAS+ goblet/mucous cells per mm basal lamina (BL) in CS-exposed macaques compared to FA group (**Figure 1B**). The expression levels of secretory mucin *MUC5AC* mRNA were 3-fold higher in CS group compared to FA macaques (**Figure 1C**) as determined by qRT-PCR. CS-induced MUC5AC expression was also corroborated by immunostaining of bronchial tissue sections (**Figure 1D**) with 7.9-fold higher percentage of MUC5AC-positive (MUC5AC^+^) cells observed in CS macaques over FA group (**Figure 1E**). Thus, chronic CS exposure results in goblet cell hyperplasia with increased expression of MUC5AC mucin.

LncRNAs are essential regulators of smoke mediated inflammatory responses (6, 7) and based on the critical role of *LASI* lncRNA in airway inflammatory and mucus hyperexpression (11), we analyzed the effects of CS on bronchial epithelial *LASI* lncRNA expression. Compared to the FA group, we observed a 2.4-fold increase in *LASI* transcript levels in CS-exposed macaques (**Figure 1F**). Cellular *LASI* expression levels were further analyzed by performing RNA-FISH as described previously (11), which allowed for single RNA molecule-level resolution and subcellular localization evaluation (**Figure 1G**). We found that the number of *LASI* lncRNA transcripts per cell was significantly upregulated upon CS treatment (**Figure 1H**). Overall, in a large animal model of chronic CS exposure, bronchial epithelial cells show a strong correlative increase in *LASI* lncRNA levels and MUC5AC mucin expression.

### Disease Severity Associated Increased Mucin Expression, Inflammation, and Mucous Cell Hyperplasia in COPD Airways

**Figure 2:**
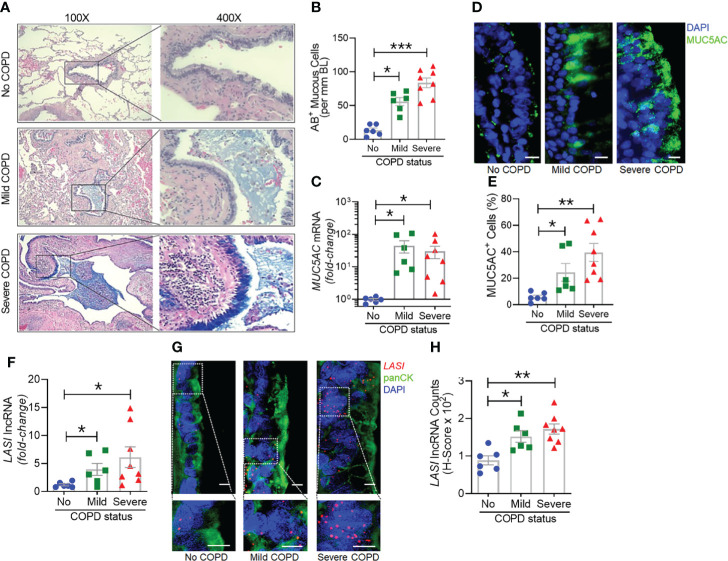
Archived airway sections of COPD patients show increased mucus expression and goblet cell hyperplasia along with increased *LASI* lncRNA expression in epithelial cells. **(A)** Representative histomicrographs of airway tissue sections from subjects with No, Mild, or Severe COPD stained with alcian blue (AB). Images are shown at 100x magnification and inset images are magnified 400x in the right panels. **(B)** Quantification of AB+ mucous/goblet cells per mm of basal lamina (BL). **(C)** Relative quantities of *MUC5AC* mRNA levels in tissues from mild and severe COPD subjects compared to control subjects with no COPD, analyzed by qRT-PCR. **(D)** Micrographs showing MUC5AC mucin immunopositivity (green) in tissue sections and counterstained with DAPI (shown in blue) to identify nuclei, scale – 10 µm. **(E)** Quantification of MUC5AC+ cells per mm BL in each group. **(F)** Quantitation of *LASI* lncRNA levels in mild and severe COPD subjects compared to control subjects with No COPD. **(G)** Micrographs showing *LASI* lncRNA levels in airway epithelial cells of patient bronchial tissues. *LASI* lncRNAs were detected by RNA-FISH (shown in red) and epithelial cells were immunostained by pan-cytokeratin (panCK, shown in green) antibody, and nuclei (shown in blue) were stained by DAPI. Lower panels show magnified images of the insets drawn in upper panels (scale – 5µm). **(H)** Quantitation for *LASI* lncRNAs per epithelial cell as measured by H-score analysis. Data shown as mean ± SEM; n=6-8/gp; data analyzed by ANOVA with multiple comparisons; *p<0.05; **p<0.01; ***p<0.001.

Next, we analyzed the archived lung tissue samples obtained from 14 COPD patients and 6 control donors with no-COPD (**Table 1**). All the samples were from former smokers with or without COPD were stratified based on the NIH and WHO’s Global Initiative for Chronic Obstructive Lung Disease (GOLD) criteria (17). According to these criteria, six donor patients had mild (GOLD stage 1 or 2) COPD, and eight donor patients had severe (GOLD stage 3 or 4) COPD. To evaluate airway mucoglycoproteins (mucins) and mucous/goblet cells, tissue sections were stained with AB-H&E as described (11). Among the severe COPD patients, the small airways had disproportionately prominent mucus masses or plugs (**Figure 2A**). Based on the number of AB+ mucous cells in each group, there was a significant increase in mucus expressing cells in severe COPD tissue samples (**Figure 2B**). Specifically, there were 31.6, 82.9, and 113.4 mucous cells per mm BL in no-COPD, mild-COPD, and severe-COPD samples, respectively. Mucus hypersecretion is a driving factor of COPD pathology and CS exposure induces the of secretory mucin MUC5AC levels (17–21). We also found that *MUC5AC* mRNA levels were significantly upregulated in mild and severe COPD tissue samples, with 44- and 30-fold higher expression, respectively, compared to no-COPD control tissues (**Figure 2C**). In addition, we immunoprobed the tissue samples for MUC5AC protein expression and both mild and severe COPD samples showed higher MUC5AC immunopositivity (**Figure 2D**). Compared to no-COPD controls, there was a 4.2-fold and 6.8-fold increase in the percentage of MUC5AC+ cells in mild and severe COPD samples, respectively (**Figure 2E**). However, there was no significant change in the expression levels of a master transcriptional regulator of mucus response, *SPDEF* (**Supplementary Figure S1A**). Among inflammatory factors, mRNA levels of *IL-6* were upregulated in mild COPD samples only (**Supplementary Figure S1B**) and *ICAM-1* mRNAs showed a trend towards upregulated expression in mild and severe COPD tissue samples, as compared to no-COPD controls (**Supplementary Figure S1C**). These data corroborate the association of small airway tissue pathology with clinically defined COPD disease status and increased mucoinflammatory responses among COPD subjects.

**Table 1:** Demographics of the study cohort of COPD patients with clinically-defined GOLD stage severity and self-reported smoking history.

	No COPD	Mild COPD	Severe COPD
Age*	54.2±3.2	69.2±3.9	65.1±2.7
Gender, M/F	3M/3F	4M/2F	3M/5F
Smoking in PY*	54.0±12.6 (2)	29.9±10.7 (3)	35.0±2.8 (3)
Stop Smoking (Y)*	6.6±1.6	21.4±12.8	11.9±2.5

^*^Mean±SEM; M, Male; F, Female; PY, Packs per Year; Y, Years.

### *LASI* lncRNA Levels Are Upregulated in COPD Airway Epithelium

We next investigated the correlation between the airway epithelial *LASI* lncRNA expression and the mucoinflammatory phenotype of COPD tissue samples. The transcript expression levels in lung tissue homogenates of COPD and no COPD control samples were evaluated by qRT-PCR. *LASI* transcript levels were 4-fold and 6-fold higher in the mild COPD (n=6) and severe COPD tissue samples, (n=8), respectively, compared to no-COPD control tissues (n=6) (**Figure 2F**). We also investigated of the expression of other lncRNAs such as, ICAM-1-related lncRNA (*ICR*), which regulates ICAM-1 expression by mRNA stabilization *via* direct interaction and duplex formation (22), but there was no significant change in ICR levels among COPD tissue samples (**Supplementary Figure S1D**). Similarly, there was no change in the expression levels of highly prevalent lncRNA *NEAT1* or nuclear enriched assembly transcript 1 (**Supplementary Figure S1E**) and *MALAT1* or metastasis associated lung adenocarcinoma transcript 1 (**Supplementary Figure S1F**). *NEAT1* lncRNA has been implicated as a potential prognostic marker of COPD exacerbations where its expression level correlated with disease severity (23). Similarly, *MALAT1* lncRNA has been proposed as a potential therapeutic target because silencing its expression blocked the COPD-associated lung remodeling (10). We also analyzed the expression levels of lncRNA called *WAKMAR2* or a wound and keratinocyte migration-associated lncRNA, which regulates the proinflammatory responses in keratinocytes (24) and there were no significant changes among COPD subjects (**Supplementary Figure S1G**).

To further corroborate the COPD-associated *LASI* lncRNA expression levels and to evaluate airway epithelium specific expression, we conducted RNA-FISH to examine *LASI* expression at a single RNA molecule resolution and evaluate the *LASI* subcellular localization (**Figure 2G**). We found that in small airways, both mild and severe COPD tissue samples present with significantly upregulated *LASI* expression as compared to non-COPD controls. There was 1.4- and 1.6-fold higher *LASI* expression in pan-cytokeratin+ epithelial cells of mild and severe COPD tissues, respectively (**Figure 2H**). *LASI* transcripts were found both in the perinuclear and the cytosolic regions of bronchial epithelial cells. Collectively, these data suggest a strong correlation between *LASI* lncRNA expression and mucus hypersecretion, mucous cell hyperplasia, and COPD pathogenesis.

### Primary HBECs From COPD Patients Show Higher Transcript Levels of *LASI* lncRNA and Mucoinflammatory Factors

**Figure 3:**
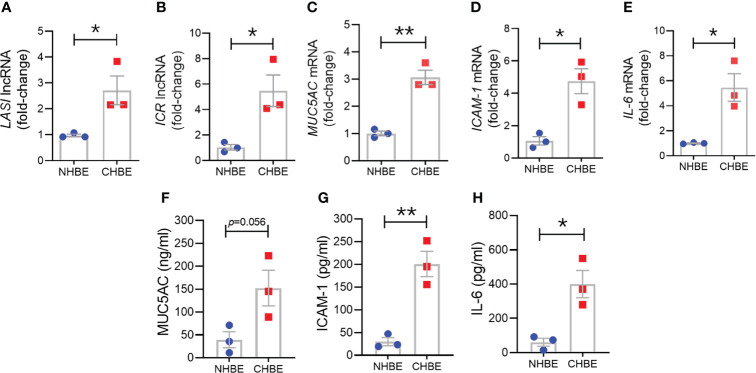
Differentiated bronchial epithelial cells from COPD subjects show increased expression of immunomodulatory lncRNAs, MUC5AC mucin, and IL-6 and ICAM-1 compared to control cells from non-COPD donors. Relative transcript levels for lncRNA *LASI* **(A)**, and *ICR* **(B)** in 3D cultured and unstimulated NHBE and CHBE cells as determined by qRT-PCR. Relative mRNA levels of *MUC5AC* **(C)**, *ICAM-1* **(D)**, and *IL-6* **(E)** inflammatory factors. Quantification of secretory MUC5AC mucin levels **(F)** in the apical washes, and the secreted ICAM-1 **(G)**, and IL-6 **(H)** levels in basal media supernatant as analyzed by specific sandwich ELISA assays. Data shown as mean ± SEM as fold-change over NHBEs; n = 3/gp; data analyzed by student’s t-test; *p<0.05; **p<0.01.

To assess epithelial cell-specific responses, we next cultured primary differentiated human bronchial epithelial cells (HBECs) obtained from COPD donors (CHBEs) and compared with HBECs from control donors with no-COPD (NHBEs). Primary NHBEs and CHBEs were differentiated on air-liquid interface (ALI), as 3D airway cultures. We first compared the baseline differences between differentiated NHBEs and CHBEs, without any treatment or stimulation. Among the lncRNAs analyzed, expression levels of ICAM-1 loci associated lncRNAs, *LASI* (**Figure 3A**) and *ICR* (**Figure 3B**) were 6.2- and 8.0-fold higher in unstimulated CHBEs compared to NHBEs, respectively. Expression levels of other lncRNAs were also higher in CHBEs with *NEAT1*, *MALAT1*, and *WAKMAR-2* lncRNAs expressed at 3.3-, 1.6-, and 3.2-fold higher in CHBEs compared to NHBEs, respectively (**Supplementary Figures S2A–C**).

Next, we examined the baseline expression of epithelial inflammatory factors and secretory mucins in NHBEs and CHBEs. Compared to NHBEs, the *MUC5AC* mucin (**Figure 3C**), *ICAM-1* (**Figure 3D**), and *IL-6* (**Figure 3E**) mRNA levels were 3.0-, 4.6-, and 6.0-fold higher in CHBEs. In order to determine whether the changes in transcript levels recapitulate the secretory protein levels, we analyzed the MUC5AC protein levels in apical wash samples and found that MUC5AC levels were approximately 39.1 ng/ml and 152.2 ng/ml in NHBEs and CHBEs, respectively (**Figure 3F**). Protein levels of ICAM-1 and IL-6 were analyzed in NHBE and CHBE basal culture media supernatants. Secreted ICAM-1 levels were approximately 29.9 pg/ml and 200.8 pg/ml in NHBE and CHBE culture media, respectively (**Figure 3G**), with a 6.7-fold increase in CHBEs. Similarly, average secreted IL-6 level in NHBE culture media was 60.6 pg/ml whereas in CHBE culture media was 399.8 pg/ml (**Figure 3H**). Collectively, these data suggest a strong dysregulation of inflammatory responses in the bronchial epithelial cells in COPD with coordinated changes in lncRNA, mRNA, and protein expression.

### CS Exposure Results in an Augmented Inflammatory Response in COPD HBECs

**Figure 4:**
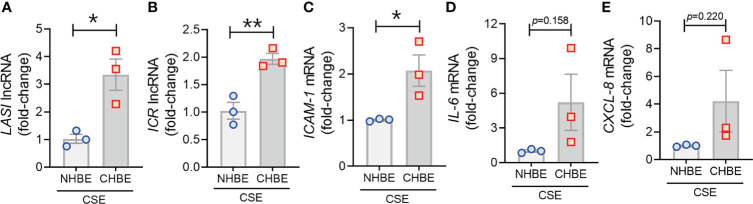
Cigarette smoke exposure of COPD bronchial epithelial cells induces higher levels of immunomodulatory lncRNAs, and inflammatory factor mRNAs compared to non-COPD control cells. Primary NHBEs and CHBEs grown in submerged culture setting were treated with a 20 µg/ml cigarette smoke extract (CSE) and forty-eight hours after treatment cells were harvested and qRT-PCR was performed. Relative transcript levels for lncRNA *LASI* **(A)**, and *ICR* **(B)** in CSE-treated NHBE and CHBE cells as determined by qRT-PCR. Relative mRNA levels of *ICAM-1* **(C)**, *IL-6* **(D)**, and *CXCL-8* **(E)** inflammatory factors. Data shown as mean ± SEM as fold-change over NHBEs; n=3/gp; data analyzed by student’s t-test; *p<0.05; **p<0.01.

To evaluate the CS-induced response, CHBEs and NHBEs were treated with 20 μg/ml CSE for 48h as described previously (25), and total cell RNA was evaluated for changes in lncRNAs and inflammatory factors’ expression. Interestingly, both ICAM-loci associated lncRNAs, *LASI* (**Figure 4A**) and *ICR* (**Figure 4B**) were significantly upregulated with 3.3-fold and 1.9-fold upregulation in CSE-treated CHBEs, respectively, as compared to CSE-treated NHBEs. However, expression levels of *NEAT1* and *MALAT1* lncRNAs failed to show any significant change following CSE treatment (**Supplementary Figures S3A, B**), but the *WAKMAR-2* lncRNA levels were 2.8-fold higher following CSE-treatment of CHBEs over NHBEs (**Supplementary Figure S3C**). This change in CSE-induced lncRNAs directly correlated with expression of *ICAM-1* mRNA, which was 2.0-fold upregulated in CSE-treated CHBEs than NHBEs (**Figure 4C**). No significant changes were observed in CSE-induced *IL-6* and *CXCL-8* mRNA expression between CHBEs and NHBEs (**Figures 4D, E**). These data suggest that the CSE induces a dysregulated response in NHBEs, and the responses are further potentiated in CHBEs; and that there is a potential direct regulatory relationship between *LASI* and *ICR* lncRNAs and ICAM-1 expression.

**Figure 5:**
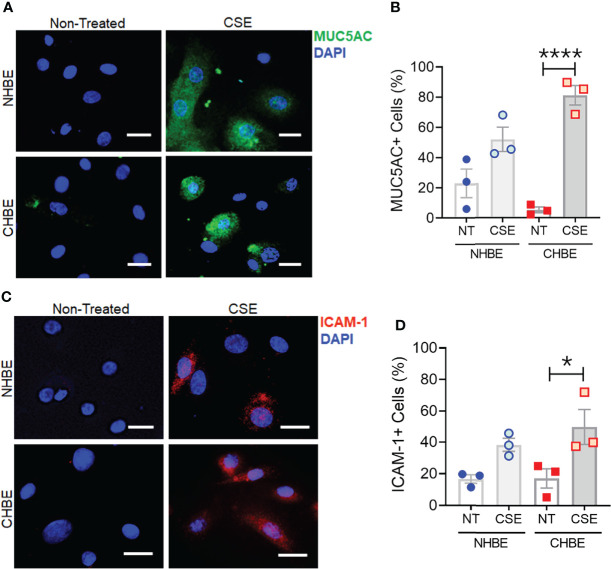
Cigarette smoke treatment augments mucous cell hyperplasia in CHBEs with higher ICAM-1 protein expression compared to non-COPD control cells. Primary NHBEs and CHBEs grown in LabTek-II^®^ slides were treated with a 20 µg/ml CSE and forty-eight hours after treatment cells were fixed with 4% paraformaldehyde (PFA) and processed for staining with antibodies against MUC5AC and ICAM-1. **(A)** Representative micrographs showing MUC5AC immunopositivity (shown in green) in NHBEs and CHBEs treated with CSE or left non-treated (NT), and nuclei were stained by DAPI (shown in blue), scale – 10µm. **(B)** Quantification of MUC5AC-positive (+) cells in NHBEs and CHBEs treated with CSE compared to NT cells. **(C)** Representative micrographs showing ICAM-1 immunopositivity (shown in red) in NHBEs and CHBEs treated with or without CSE, scale – 10 µm. **(D)** Quantification of ICAM-1-positive (+) cells in NHBEs and CHBEs treated with CSE compared to NT controls. Data shown as mean ± SEM as fold-change compared to NT cells; n=3/gp; data analyzed by ANOVA; *p<0.05; ****p<0.0001.

To further substantiate these findings, we additionally performed cytometric analysis of MUC5AC and ICAM-1 protein expression in NHBEs and CHBEs grown on Labtech^®^ slides and treated with 20µg/ml CSE for 48h. The cells showing immunopositivity for MUC5AC (MUC5AC+) and ICAM-1 (ICAM-1+) were quantified and there was a 2.3-fold increase in MUC5AC+ cells in CSE-treated NHBEs, and a 15.4-fold increase in CSE-treated CHBEs (**Figures 5A, B**). Similarly, NHBEs showed a 2.3-fold increase in ICAM-1+ cells, while CHBEs showed a 2.9-fold increase in ICAM-1+ cells upon CSE treatment (**Figures 5C, D**). These data further suggest that CS insult causes CHBEs to respond with a significantly more severe, dysregulated mucus secretory and inflammatory response.

### Knocking Down *LASI* Expression Suppresses the Smoke-Induced Inflammation, Mucin Expression, and Mucus Cell Hyperplasia

**Figure 6:**
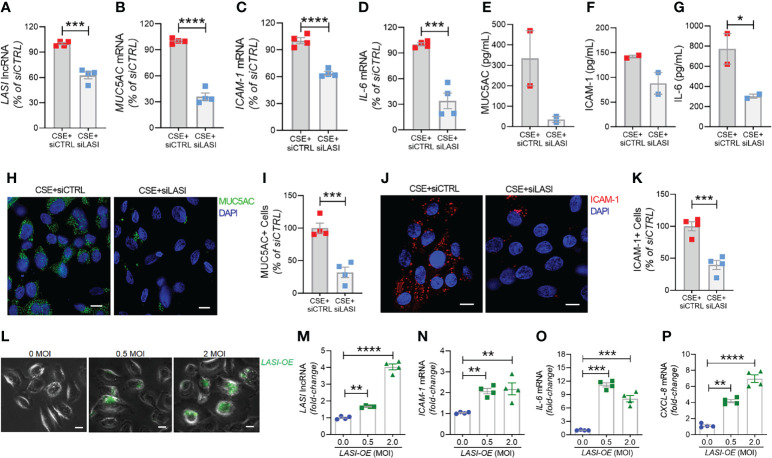
Silencing *LASI* lncRNA suppresses the CSE-induced mucoinflammatory responses whereas *LASI* overexpression augments the expression of inflammatory factors. CHBEs grown in 3D ALI tissue culture conditions were transfected with either siRNA targeting *LASI* (siLASI) or scrambled control siRNA (siCTRL), and cells were treated with 20 µg/ml CSE to obtain CSE+siLASI and CSE+siCTRL cells, respectively. Cells harvested forty-eight hours post CSE-treatment were analyzed for the expression levels of *LASI* lncRNA **(A)**, and mRNA levels of *MUC5AC* **(B)**, *ICAM-1* **(C)**, and *IL-6* **(D)** by qRT-PCR. Apical washes from 3D tissue cultures were analyzed for MUC5AC mucin **(E)** by ELISA, and the basal media supernatants were analyzed for ICAM-1 **(F)**, and IL-6 **(G)** protein levels by specific ELISA assays. **(H)** Representative micrographs of CHBEs transfected with siCTRL or siLASI and treated with CSE showing MUC5AC mucin immunopositivity (shown in green) and nuclei were stained by DAPI (shown in blue), scale – 5µm. **(I)** Quantification of MUC5AC+ cells among CSE+siLASI CHBEs, shown as percentage of CSE+siCTRL cells. **(J)** Micrographs of CHBEs showing ICAM-1 immunopositivity (shown in red), scale – 5 µm. **(K)** Quantification of ICAM-1+ CHBEs. Data shown as mean ± SEM compared to CSE+siCTRL cells (n=4); data analyzed by student’s t-test; *p<0.05; ***p<0.001; ****p<0.0001. **(L)** Representative micrographs of cells transduced with 0, 0.5, and 2 MOI of LASI-OE lentiviral preparation showing GFP reporter fluorescence (shown in green) and phase contrast images of cells, scale – 5µm. Cells harvested forty-eight hours post-transduction were analyzed for expression levels of *LASI* lncRNA **(M)**, and mRNA levels of *ICAM-1* **(N)**, *IL-6* **(O)**, and *CXCL-8* **(P)** by qRT-PCR. Data shown as mean ± SEM compared to mock-transduced cells (0 MOI); data analyzed by ANOVA with multiple comparisons; **p<0.01; ***p<0.001; ****p<0.0001.

In order to determine whether the correlation between *LASI* lncRNA with *MUC5AC* and *ICAM-1* expression is functionally significant, we genetically silenced *LASI* lncRNA expression using siRNAs targeting *LASI* (siLASI), as described previously (11), in differentiated CHBEs then challenged with 20 μg/ml CSE for 48h. CHBEs transfected with negative control siRNA (siCTRL) followed by 48 h 20 μg/ml CSE treatment served as controls. Compared to siCTRL, the siLASI-transfected CHBEs showed a 37.5% reduction in *LASI* lncRNA expression (**Figure 6A**). Interestingly, even with this moderate reduction in *LASI* expression, there was a 63.9% reduction in CSE-induced *MUC5AC* mRNA levels in siLASI-transfected CHBEs (**Figure 6B**), suggesting that functional availability of *LASI* lncRNA is necessary for CSE-mediated induction of *MUC5AC* expression in CHBEs. Notably, we found no change in *SPDEF* transcription factor levels (**Supplementary Figure S4A**), suggesting that a CSE-induced and *LASI*-mediated MUC5AC expression may not be dependent on SPDEF-mediated transcriptional upregulation. Expression levels of another airway secretory mucin, *MUC5B* mRNA were not changed in siLASI-transfected CHBEs (**Supplementary Figure S4B**). We also evaluated the changes in the CSE-mediated inflammatory responses in siLASI-transfected CHBEs. Notably, we found that siLASI induced a significant 36.2% reduction in *ICAM-1* mRNA levels (**Figure 6C**) and a 66% reduction in *IL-6* mRNA levels (**Figure 6D**), suggesting that *LASI* lncRNA may contribute to the expression of these inflammatory factors. However, expression levels of inflammatory factor CXCL-8 mRNAs were not changed in siLASI-transfected CHBEs (**Supplementary Figure S4C**).

Next, we used sandwich ELISA to measure the secreted protein levels of mucin MUC5AC, and cytokines ICAM-1 and IL-6 in siLASI-transfected CHBEs followed by 48h CSE treatment. Interestingly, apical wash from siCTRL-transfected CHBEs had 236.6 ng/ml of mucin MUC5AC while the siLASI-transfected CHBEs had 73.9 ng/ml (**Figure 6E**). Furthermore, the culture media supernatants from the siLASI-transfected CHBEs had 298.2 pg/ml IL-6 levels compared to the 765.8 pg/ml in siCTRL-transfected CHBEs (**Figure 6F**), Similarly, siLASI-transfected CHBEs secreted 105.3 pg/ml ICAM-1 levels whereas the siCTRL-treated CHBEs secreted 138.3 pg/ml (**Figure 6G**). Overall, siLASI-transfected CHBEs showed a 2.6-fold reduction in IL-6 and a 1.3-fold reduction in ICAM-1 secretory levels following CSE treatment over siCTRL-transfected CHBEs. Notably, CSE-induced MUC5AC mucin secretion was reduced by 3.2-fold in siLASI-transfected CHBEs compared to the siCTRL-transfected CHBEs.

We further corroborated the data by immunoprobing the siCTRL- and siLASI- transfected CHBEs for MUC5AC and ICAM-1 protein expression following 48 h CSE treatment (**Figures 6H, J**). We found that silencing LASI expression by siLASI resulted in a 3.1-reduction in MUC5AC-expressing (MUC5AC+) cells (**Figure 6I**) and a 2.5-fold reduction in cell expressing ICAM-1 protein (**Figure 6K**). These data strongly suggest lncRNA *LASI* represents an important regulatory mediator in the CS-induced pathophysiological changes observed in COPD airways, including dysregulated immune response and chronic mucus hypersecretion.

### Ectopic *LASI* lncRNA Expression Results in Increased Expression of Inflammatory Factors

To determine whether *LASI* lncRNA directly mediates the expression of inflammatory factors, a lentiviral preparation encoding *LASI* lncRNA was used to transduce airway epithelial cells, and the ectopic *LASI* overexpression (LASI-OE) was followed by assessing GFP-tag fluorescence (**Figure 6L**). Compared to mock-transduced (0 MOI) controls, cells transduced with 0.5 and 2.0 MOI of lentivirus-*LASI* resulted in 1.7- and 4.0-fold increased expression of *LASI* lncRNA, respectively (**Figure 6M**). Set of control cells transduced with empty vector lentiviral preparation (Lenti-EV) showed no change in the expression of airway *LASI* lncRNA or associated inflammatory factor mRNAs (**Supplementary Figure S5**). Interestingly, LASI-OE cells showed two-fold or higher levels of *ICAM-1* mRNA expression (**Figure 6N**) and expression levels of *IL-6* (**Figure 6O**) and *CXCL-8* (**Figure 6P**) mRNAs were also increased by as much as eight- and four-fold, respectively. Thus, ectopic overexpression of *LASI* lncRNA directly upregulates the airway epithelial inflammatory factor mRNA levels.

## Discussion

**Figure 7:**
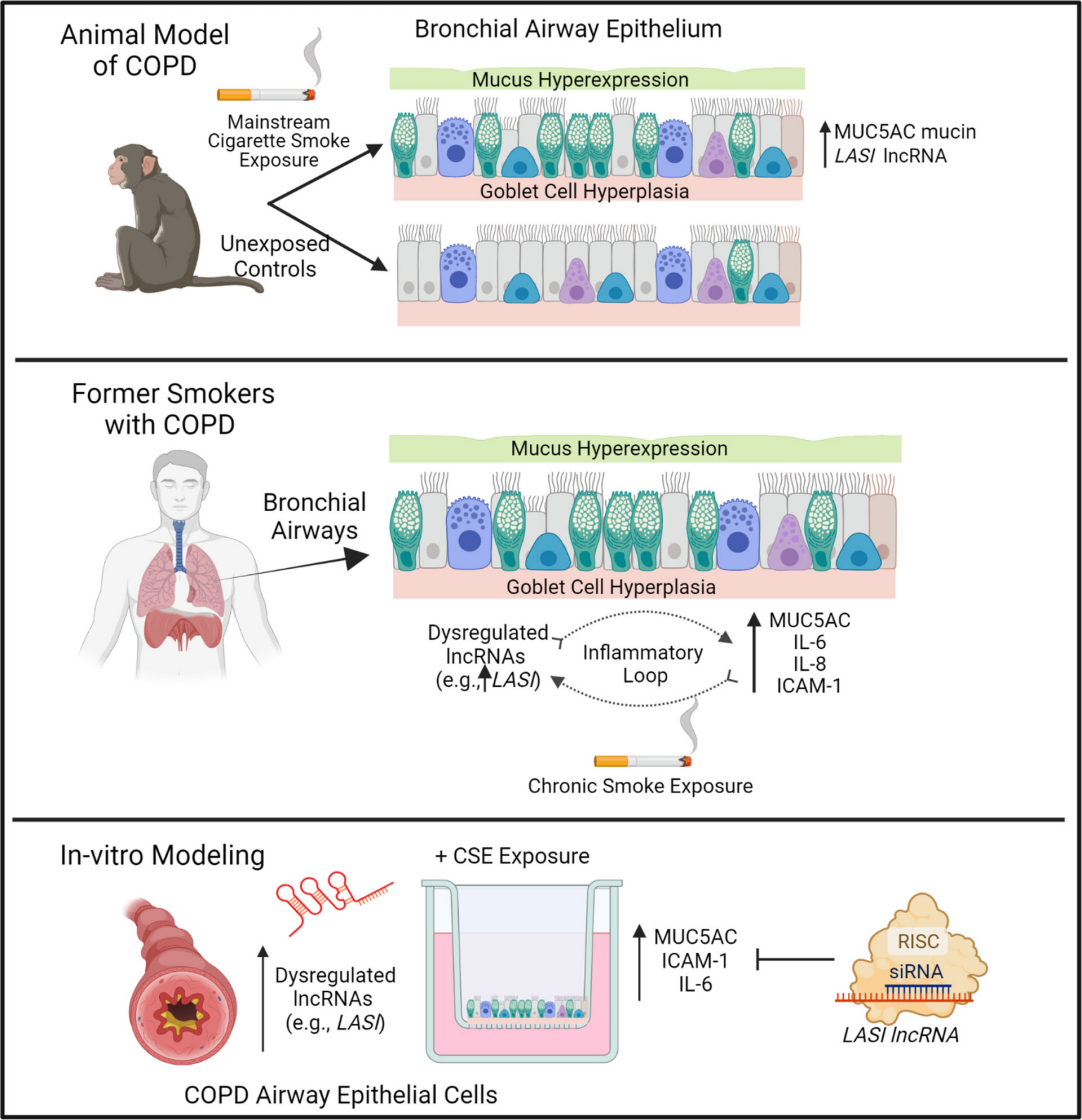
Schematic representation on the potential role of bronchial epithelial LASI lncRNA in CS-induced and COPD-associated airway mucus hyperexpression and inflammatory responses.

CS exposure is the most important and the best-studied risk factor associated with COPD. That said, studies are needed to unravel novel underlying molecular pathways for improved diagnostic and therapeutic interventions for CS-induced pathologies and COPD-related comorbidities. The newly discovered lncRNA molecular species are now being proposed as novel cellular entities that play an important role in physiology and pathophysiology (6, 26). We have recently identified novel immunomodulatory lncRNAs that may play a crucial role in airway inflammatory responses. In the present study, we find a strong association of immunomodulatory *LASI* lncRNA expression with CS-induced airway mucus hyperexpression and inflammatory responses as summarized in **Figure 7**. The correlation was observed both in a large animal model of CS-induced COPD, and in lung tissue samples from former smokers with COPD in comparison with tissues from former smokers with no COPD. We found that lung tissue samples of CS-exposed macaques and those of COPD patients (with mild and severe COPD) presented with an increased *LASI* lncRNA expression in bronchial airway epithelial cells. *LASI* lncRNA expression correlated to the increased expression of secretory mucin MUC5AC, and innate airway inflammatory factors, IL-6, and ICAM-1, which were all upregulated in COPD tissue samples and in macaques exposed to mainstream CS. These data suggested that *LASI* lncRNA may play a role in smoke-associated bronchial epithelial remodeling and COPD. To validate the airway epithelial specific significance of *LASI* lncRNA in CS-induced responses, we utilized a 3D airway tissue culture model of primary HBECs from COPD and compared the baseline and CSE-induced responses to that of control cells from donors with no COPD. We found that unstimulated CHBEs, show a baseline upregulation of *LASI* lncRNA, along with other immunomodulatory lncRNAs such as *ICR*, *NEAT1*, *MALAT1*, and *WAKMAR2*. However, none of these lncRNAs were responsive to CSE-treatment except for *LASI* and *ICR* lncRNAs. This led us to explore the responses to CS exposure and *LASI* was the only lncRNA explored in this report which showed a potentiated dysregulated response to CSE treatment in CHBEs as compared to NHBEs. *LASI* lncRNA further showed a strong correlation with expression levels of ICAM-1, as well as IL-6 and CXCL-8, suggesting a functional importance in COPD pathogenesis. Accordingly, we knocked down *LASI* lncRNA expression in CHBEs and discovered that a reduction in *LASI* lncRNA expression resulted in a significant reduction in CSE-induced mucoinflammatory response by reducing the expression of MUC5AC, IL-6, and ICAM-1 levels both at mRNA and protein levels. These data thus collectively implicate *LASI* lncRNA as a novel mediator of CS-induced and COPD-associated airway pathophysiologies. Moreover, even in the absence of any external stimulation, the ectopic overexpression of *LASI* lncRNA directly upregulated the transcript levels of airway inflammatory factors, suggesting that *LASI* lncRNA may be directly involved in the transcriptional upregulation or mRNA stability of these inflammatory factors.

Cigarette smoking is strongly associated with COPD where more than 50% of COPD patients are active smokers and over 70% have a history of smoking. Notably, over 50% of COPD mortality is attributable to active smoking (27). Patients who are exposed to CS present with exacerbated mucus secretory and airway inflammatory conditions. As such, the dysregulated response to smoke exposure may provide the most useful insight into the severe COPD pathology and potentially fatal exacerbations (28, 29). However, due to limitation of the longitudinal sampling from human COPD subjects and the other genetic and environmental heterogeneity, animal models of COPD following chronic CS exposure are widely studied to understand the smoke-mediated disease pathogenesis. Small animal models of CS exposure do not recapitulate all aspects of human COPD specifically in bronchial airway remodeling and therefore, we recently performed a study using the cynomologus macaque model of chronic CS exposure. Following 27-weeks of mainstream CS exposure, these animals show reduced lung functions and chronic bronchitis similar to that observed in COPD smokers compared to control macaques kept in room air. Thus, we were able to employ the archived lung tissues from these well-characterized macaques to establish the correlation of CS-induced airway inflammatory responses with airway lncRNA expression. Due to the labor- and time-intensive nature of these large animal model studies, we are currently analyzing whether there is a murine homolog of *LASI* lncRNA or if there is any other airway-specific lncRNA of rodent airways involved in CS-mediated inflammation. Accordingly, future studies will be planned to test the *in-vivo* efficacy of targeting lncRNAs in suppressing CS-mediated airway remodeling and mucoinflammatory responses.

Airway mucus hypersecretion is the hallmark of COPD pathogenesis, enabling the compounding cascade of inflammation, ROS generation, and airborne pathogen retention in airways due to compromised mucociliary clearance, distal airway occlusion and inability to effectively clear the airways (4). Airway mucins MUC5AC and MUC5B are the predominant gel-forming mucins in COPD, and CS exposure and frequent bacterial or viral infection synergistically amplify MUC5AC levels (17–21). Several other inflammatory biomarkers have been implicated with COPD and smoke-associated exacerbations (26). Among the most prominent factors are the ICAM-family proteins, specifically ICAM-1, and an innate inflammatory cytokine, IL-6 which have shown strong association to decreased lung function in COPD, both in active and former smokers with varying degree of severity (30). Airway epithelial cells play a vital role in the secretion of ICAM-1 and IL-6, and could serve as drivers of the chronic changes observed in COPD (31).

Till date, thousands of lncRNAs have been discovered, however, studies on the functional significance of the changes in lncRNA expression are lacking. The structural flexibility and 3D-conformation enables lncRNAs to interact with large number of cellular macromolecules including proteins, DNA, RNA, and chromatin to modulate epigenetic and transcriptomic changes and associated cellular responses (8). Large number of lncRNAs have been implicated in pathology, including chronic pulmonary conditions, CS-related immune responses, and inflammatory regulation (6, 9, 10). LncRNA-mediated regulation of innate immune responses is potentially central for the establishment of host-beneficial trained immunity (32), but these responses could be dysregulated in case of chronic pulmonary disease resulting in hyperactive inflammatory responses. Microarray analysis has shown over 39,000 lncRNAs are differentially expressed in COPD patients, stratified by smoking status (33). Numerous lncRNAs have been experimentally characterized and shown to affect the inflammatory responses of airway epithelium *via* epigenetic and/or transcriptomic mechanisms and induce an accelerated aging of lung epithelium associated with COPD (6).

Goblet cell hyperplasia is a salient feature of COPD pathology where the 33% of distal conducting airway epithelium is comprised of goblet cells in COPD lungs, versus less than 5% observed in the distal airways of non-COPD lungs (34). We also report a disease severity associated increase in goblet cell hyperplasia in COPD tissue samples, with a 2.1- to 3.0-fold increase in goblet cell numbers per mm BL in mild and severe COPD tissues, respectively. This suggests accurate modeling of molecular level changes in COPD epithelium. We found that the lncRNA *LASI* correlates with disease severity, while other lncRNAs do not, suggesting a potential regulatory role which we further explored using an *in vitro* COPD model. Our panel of lncRNAs showed the *NEAT1*, *MALAT1* and *WAKMAR2* were not upregulated following CSE- treatments. Interestingly, Hu and colleagues (2020) reported increased expression of MALAT1 in COPD lung tissue specimens, however we found that there was no change in MALAT1 expression in correlation with disease severity (10). However, to evaluate whether the effects observed were specifically driven by CS exposure, we relied on the archived tissue samples from C. macaques that were exposed to CS chronically and the data strongly suggests that long-term CS exposure is a driving factor behind the airway pathology observed. Importantly, we used primary HBECs from controls with no COPD and with COPD i.e., NHBEs and CHBEs for *in-vitro* validation of our ex-vivo findings. Recent reports have shown that the pathologic changes in epithelial histology, goblet cell numbers, and mucus hypersecretion are preserved in differentiated COPD subject-derived lung epithelial cells in ALI culture settings, and transcriptomic analysis showed over 200 differentially expressed transcripts (35). The ciliary beating impairment has also been found to be reflected in differentiated CHBEs versus NHBEs. Here, we report a significant higher baseline expression, of MUC5AC, ICAM-1, and IL-6 in CHBEs, as compared to NHBEs without any treatment or stimulation. Thus, our *in-vitro* model do recapitulates the differences observed in COPD subjects thus suggesting of epigenetic and transcriptomic transformations that are preserved in bronchial epithelial cells. Further, CHBEs also showed a significantly increased baseline levels of *LASI* lncRNA as compared to NHBEs. In addition, there was an increased expression of *ICR*, *NEAT1*, *MALAT1*, and *WAKMAR2* lncRNAs in cultured CHBEs, however, we did not observe any significant change in these lncRNAs in lung tissue homogenates of COPD subjects. This suggests that these lncRNAs may respond in cell/tissue-context manner, and the epithelial expression of these lncRNA may not play a direct role in COPD pathogenesis.

Also of note, we found that blocking *LASI* lncRNA expression in CHBEs led to a suppressed induction of MUC5AC mucin expression with no change in transcription factor SPDEF expression, suggesting that CSE-mediated mucin expression may not directly involve the previously observed *LASI* lncRNA-and SPDEF-mediated mucin upregulation observed in allergic asthma studies (11). CSE exposure induces SPDEF *via* NOTCH3 signaling, suggesting that *LASI*-mediated dysregulation is not dependent on NOTCH3, and further suggests a need to evaluate the role of *LASI* lncRNA on other signaling pathways involved in CS-mediated mucoinflammatory responses such as epithelial growth factor receptor (EGFR)-mediated inflammation (36). EGFR-mediated signaling is likely to be the predominant driver of airway remodeling and mucus cell hyperplasia in CS-induced COPD pathogenesis, and this pathway may not involve SPDEF-mediated mucous responses (11, 37). In terms of molecular regulation of other inflammatory factors' expression, lncRNAs are shown to act as molecular sponges/scaffolds for miRNAs as reported recently in COPD (38–40). For example, lncRNA TUG1 promotes airway remodeling *via* suppressing the miR-145-5p in CS-induced COPD models. The lncRNA NNT-AS1 was shown to regulate COPD associated airway cell proliferation/cell death, inflammation, and remodeling *via* the miR-582-5p and FBXO11 pathways. Interestingly, high levels of IL-6 and lncRNA IL6-AS1 were reported in COPD subjects with concurrent upregulation of miR-149-5p and early B-cell factor 1. Similar studies are underway to determine possible *LASI* lncRNA binding partners using the LASI-OE approach described in the present study to identify the molecular mechanisms responsible for plausible increased transcriptional activity or mRNA stability that augments the airway inflammatory responses.

LncRNAs modulate gene expression at multiple levels to alter the cell functions/responses. They are known to modulate chromatin structure or bind to directly to DNA. They can also bind to and suppress the expression of miRNAs or pre-miRNAs (8, 41). LncRNAs can directly enhance or suppress the expression of many mRNAs or functional transcripts. In our studies, knockdown of *LASI* lncRNA led to a suppressed expression of CSE-induced *ICAM-1* and *IL-6* mRNAs suggesting that there is no direct interaction between *LASI* lncRNA and *ICAM-1* or *IL-6* mRNAs. Instead, *LASI* lncRNA may be indirectly affecting the transcription of ICAM-1 and IL-6, whereby *LASI* lncRNA may be regulating the other intermediary immunoregulatory elements such as miRNAs or promoters upstream of ICAM-1 and IL-6. Accordingly, our data posit that *LASI* lncRNA may not be directly interacting with ICAM-1 protein, mRNA, or pre-mRNA, but experimental validation are needed. In a separate study we have observed that silencing ICAM-1 expression does not affect the *LASI* lncRNA levels (data not shown), thus implicating that the expression levels of these transcripts are driven independently *via* possible mutually exclusive transcriptional regulation of the opposite strands. The data further suggests that at the transcription level, a direct induction of *LASI* lncRNA by CSE treatment may be one of the drivers for the COPD-associated mucoinflammatory responses. Furthermore, we observed that *LASI* lncRNA was expressed in perinuclear region and cytosolic regions of bronchial airway epithelial cells of both macaques and human tissues. RNA-interference based silencing works primarily in the cytosolic region and even with only 37.5% suppression of *LASI* lncRNA levels, we observed a highly significant reduction in CSE-induced MUC5AC, ICAM-1, and IL-6 expression. This data does suggest that cytosolic *LASI* lncRNA may be important mediator of mucoinflammatory response, but further cell fractionation studies are needed to determine the subcellular location specific role of *LASI* lncRNAs in driving the CSE-treatment and COPD associated inflammatory responses.

The present study has several limitations as outlined here and should be strongly considered for drawing the inferences. Firstly, the archived lung tissues from the large animal model study are from female macaques only as human epidemiological studies suggest that female smokers show higher prevalence of COPD than males but the data presented here should be interpreted accordingly. Secondly, human lung tissue samples were provided by LTRC (NIH), but the COPD patient cohort data collection relies on self-reported smoking history and lacks accuracy. Thirdly, the *in-vitro* modeling studies used NHBEs and CHBEs from three separate donors only and are from a commercial supplier with no information provided on the race, age, gender, or smoking history. Moreover, the *in-vitro* modeling used bronchial airway epithelial cells only and responses in other epithelial and submucosal cells are not investigated that could drive smoke- and COPD-associated airway remodeling. Additionally, this study is focused on *LASI* lncRNA only, which may act synergistically with additional lncRNAs, specifically with *ICR* lncRNA. Furthermore, the studies reported here used acute model of CS exposure using CS extract instead of direct mainstream smoke exposure. However, with data presented from animal model of CS exposure, and from COPD tissue and airway epithelial cells, this study does corroborate the strong association of *LASI* lncRNA with CS-induced transcriptional modulation of airway mucoinflammatory responses.

In conclusion, this study elucidates *LASI* lncRNA as a novel regulator of CSE-induced and COPD-associated airway epithelial dysregulation and further suggests that targeting *LASI* lncRNA expression could present a novel therapeutic intervention modality to treat COPD phenotypes of upper airways. Specifically, the currently available therapies have limited success in treating COPD pathophysiologies creating an unmet need in discovery of novel therapeutic avenues (42–44). Recent epidemiological and pathological studies have shown that mucus hypersecretion is a prime target of COPD treatment avenues (45). Thus, airway epithelial expressed *LASI* lncRNA may provide additional novel method of controlling mucous responses specifically when noncoding RNA-based therapeutics are shown to be promising treatment modalities (46). Present study also suggests that the lncRNA *ICR* may play a similarly important role in CHBE dysregulation and requires further investigation. Future experiments will investigate the mechanism of action of *LASI* lncRNA to identify its binding partners and potential interactions that regulate airway innate immune responses.

## Data Availability Statement

The original contributions presented in the study are included in the article/**Supplementary Material**. Further inquiries can be directed to the corresponding author.

## Ethics Statement

The animal study was reviewed and approved by Institutional Animal Care and Use Committee of Lovelace Respiratory Research Institute, Albuquerque, NM.

## Author Contributions

MM and DD designed and conducted the experiments. MM and HC wrote, edited, and/or revised the manuscript. MM, DD, and CL was responsible for data curation. HC conceptually de-signed the overall experiments and manuscript, and acquired funding. SS, MWN, GB, MNN, IR, and MS provided the oversight on the study design and edited the manuscript. All authors contributed to the article and approved the submitted version.

## Funding

The National Institutes of Health (NIH) AI159237, AI144374, HL149898, and CA241752 supported this work.

## Conflict of Interest

The authors declare that the research was conducted in the absence of any commercial or financial relationships that could be construed as a potential conflict of interest.

## Publisher’s Note

All claims expressed in this article are solely those of the authors and do not necessarily represent those of their affiliated organizations, or those of the publisher, the editors and the reviewers. Any product that may be evaluated in this article, or claim that may be made by its manufacturer, is not guaranteed or endorsed by the publisher.

## Abbreviations

AB, Alcian Blue; AB-H&E, Alcian Blue Hematoxylin and Eosin; AB-PAS, Alcian Blue and Periodic Acid Schiff’s reagent; ALI, Air-liquid interface; BL, Basal Lamina; CB, Chronic Bronchitis; CHBEs, COPD human bronchial epithelial cells; COPD, Chronic Obstructive Pulmonary Disease; CS, Cigarette Smoke; CSE, Cigarette Smoke Extract; CXCL8, C-X-C Motif Chemokine 8 or interleukin (IL)-8; FISH, Fluorescent *In-Situ* Hybridization; HBECs, Human Bronchial Epithelial Cells; ICAM-1, Intracellular Adhesion Molecule-1; *ICR*: ICAM-1-related long noncoding RNA; IL-6, Interleukin 6; *LASI*, lncRNA antisense to ICAM-1; lncRNA, Long Noncoding RNA; *MALAT1*, Metastasis Associated Lung Adenocarcinoma Transcript 1; miRNA, micro-RNA; MOI, multiplicity of infection; *NEAT1*, Nuclear Enriched Assembly Transcript 1; NHBEs, normal human bronchial epithelial cells; qRT-PCR, Quantitative Real-Time PCR; SPDEF, Sam-Pointed Domain Containing ETS Transcription Factor; TFG-β, Transforming Growth Factor β; *WAKMAR2*, Wound and Keratinocyte Migration Associated Long Noncoding RNA.

## References

[1] James SL Abate D Abate KH Abay SM Abbafati C Abbasi N. Global, Regional, and National Incidence, Prevalence, and Years Lived With Disability for 354 Diseases and Injuries for 195 Countries and Territories, 1990-2017: A Systematic Analysis for the Global Burden of Disease Study 2017. Lancet (2018) 392(10159):1789–858. doi: 10.1016/s0140-6736(18)32279-7 PMC6227754 30496104

[2] Rabe KF Watz H. Chronic Obstructive Pulmonary Disease. Lancet (2017) 389(10082):1931–40. doi: 10.1016/s0140-6736(17)31222-9 28513453

[3] Wong AWM Gan WQ Burns J Sin DD van Eeden SF. Acute Exacerbation of Chronic Obstructive Pulmonary Disease: Influence of Social Factors in Determining Length of Hospital Stay and Readmission Rates. Can Respir J (2008) 15(7):361–4. doi: 10.1155/2008/569496 PMC2679571 18949105

[4] Kim V Criner GJ. Chronic Bronchitis and Chronic Obstructive Pulmonary Disease. Am J Respir Crit Care Med (2013) 187(3):228–37. doi: 10.1164/rccm.201210-1843CI PMC4951627 23204254

[5] Vogelmeier CF Román-Rodríguez M Singh D Han MK Rodríguez-Roisin R Ferguson GT. Goals of COPD Treatment: Focus on Symptoms and Exacerbations. Respir Med (2020) 166:105938. doi: 10.1016/j.rmed.2020.105938 32250871

[6] Devadoss D Long C Langley RJ Manevski M Nair M Campos MA. Long Noncoding Transcriptome in Chronic Obstructive Pulmonary Disease. Am J Respir Cell Mol Biol (2019) 61(6):678–88. doi: 10.1165/rcmb.2019-0184TR PMC6890411 31486667

[7] Sundar IK Rahman I. Gene Expression Profiling of Epigenetic Chromatin Modification Enzymes and Histone Marks by Cigarette Smoke: Implications for COPD and Lung Cancer. Am J Physiol Lung Cell Mol Physiol (2016) 311(6):L1245–l1258. doi: 10.1152/ajplung.00253.2016 27793800 PMC5206398

[8] Schmitz SU Grote P Herrmann BG. Mechanisms of Long Noncoding RNA Function in Development and Disease. Cell Mol Life Sci (2016) 73(13):2491–509. doi: 10.1007/s00018-016-2174-5 PMC4894931 27007508

[9] Faiz A Steiling K Roffel MP Postma DS Spira A Lenburg ME. Effect of Long-Term Corticosteroid Treatment on microRNA and Gene-Expression Profiles in COPD. Eur Respir J (2019) 53(4):1801202. doi: 10.1183/13993003.01202-2018 30846474

[10] Hu TJ Huang HB Shen HB Chen W Yang ZH. Role of Long Non-Coding RNA MALAT1 in Chronic Obstructive Pulmonary Disease. Exp Ther Med (2020) 20(3):2691–7. doi: 10.3892/etm.2020.8996 PMC7401856 32765763

[11] Devadoss D Daly G Manevski M Houserova D Hussain SS Baumlin N. A Long Noncoding RNA Antisense to ICAM-1 Is Involved in Allergic Asthma Associated Hyperreactive Response of Airway Epithelial Cells. Mucosal Immunol (2021) 14(3):630–9. doi: 10.1038/s41385-020-00352-9 PMC8081750 33122732

[12] Chand HS Vazquez-Guillamet R Royer C Rudolph K Mishra N Singh SP. Cigarette Smoke and HIV Synergistically Affect Lung Pathology in Cynomolgus Macaques. J Clin Invest (2018) 128(12):5428–33. doi: 10.1172/JCI121935 PMC6264630 30277472

[13] Pauwels RA Buist AS Calverley PM Jenkins CR Hurd SS. Global Strategy for the Diagnosis, Management, and Prevention of Chronic Obstructive Pulmonary Disease. NHLBI/WHO Global Initiative for Chronic Obstructive Lung Disease (GOLD) Workshop Summary. Am J Respir Crit Care Med (2001) 163(5):1256–76. doi: 10.1164/ajrccm.163.5.2101039 11316667

[14] Han MK Agusti A Celli BR Criner GJ Halpin DMG Roche N. From GOLD 0 to Pre-COPD. Am J Respir Crit Care Med (2021) 203(4):414–23. doi: 10.1164/rccm.202008-3328PP PMC7885837 33211970

[15] Singh SP Devadoss D Manevski M Sheybani A Ivanciuc T Exil V. Gestational Exposure to Cigarette Smoke Suppresses the Gasotransmitter H(2)S Biogenesis and the Effects Are Transmitted Transgenerationally. Front Immunol (2020) 11:1628. doi: 10.3389/fimmu.2020.01628 32849552 PMC7399059

[16] Chand HS Mebratu YA Kuehl PJ Tesfaigzi Y. Blocking Bcl-2 Resolves IL-13-Mediated Mucous Cell Hyperplasia in a Bik-Dependent Manner. J Allergy Clin Immunol (2017) 140(5):1456–59.e9. doi: 10.1016/j.jaci.2017.05.038 PMC5675799 28784260

[17] Radicioni G Ceppe A Ford AA Alexis NE Barr RG Bleecker ER. Airway Mucin MUC5AC and MUC5B Concentrations and the Initiation and Progression of Chronic Obstructive Pulmonary Disease: An Analysis of the SPIROMICS Cohort. Lancet Respir Med (2021) 9(11):1241–54. doi: 10.1016/S2213-2600(21)00079-5 PMC8570975 34058148

[18] Baginski TK Dabbagh K Satjawatcharaphong C Swinney DC. Cigarette Smoke Synergistically Enhances Respiratory Mucin Induction by Proinflammatory Stimuli. Am J Respir Cell Mol Biol (2006) 35(2):165–74. doi: 10.1165/rcmb.2005-0259OC 16543607

[19] Caramori G Casolari P Di Gregorio C Saetta M Baraldo S Boschetto P. MUC5AC Expression Is Increased in Bronchial Submucosal Glands of Stable COPD Patients. Histopathology (2009) 55(3):321–31. doi: 10.1111/j.1365-2559.2009.03377.x 19723147

[20] Kesimer M Ford AA Ceppe A Radicioni G Cao R Davis CW. Airway Mucin Concentration as a Marker of Chronic Bronchitis. N Engl J Med (2017) 377(10):911–22. doi: 10.1056/NEJMoa1701632 PMC5706541 28877023

[21] Okuda K Chen G Subramani DB Wolf M Gilmore RC Kato T. Localization of Secretory Mucins MUC5AC and MUC5B in Normal/Healthy Human Airways. Am J Respir Crit Care Med (2019) 199(6):715–27. doi: 10.1164/rccm.201804-0734OC PMC6423099 30352166

[22] Guo W Liu S Cheng Y Lu L Shi J Xu G. ICAM-1-Related Noncoding RNA in Cancer Stem Cells Maintains ICAM-1 Expression in Hepatocellular Carcinoma. Clin Cancer Res (2016) 22(8):2041–50. doi: 10.1158/1078-0432.CCR-14-3106 26667486

[23] Ming X Duan W Yi W. Long Non-Coding RNA NEAT1 Predicts Elevated Chronic Obstructive Pulmonary Disease (COPD) Susceptibility and Acute Exacerbation Risk, and Correlates With Higher Disease Severity, Inflammation, and Lower miR-193a in COPD Patients. Int J Clin Exp Pathol (2019) 12(8):2837–48. PMC6949709 31934120

[24] Herter EK Li D Toma MA Vij M Li X Visscher D. WAKMAR2, a Long Noncoding RNA Downregulated in Human Chronic Wounds, Modulates Keratinocyte Motility and Production of Inflammatory Chemokines. J Invest Dermatol (2019) 139(6):1373–84. doi: 10.1016/j.jid.2018.11.033 30594489

[25] Hussain SS George S Singh S Jayant R Hu CA Sopori M. A Small Molecule BH3-Mimetic Suppresses Cigarette Smoke-Induced Mucous Expression in Airway Epithelial Cells. Sci Rep (2018) 8(1):13796. doi: 10.1038/s41598-018-32114-w 30218002 PMC6138652

[26] Nyunoya T Mebratu Y Contreras A Delgado M Chand HS Tesfaigzi Y. Molecular Processes That Drive Cigarette Smoke-Induced Epithelial Cell Fate of the Lung. Am J Respir Cell Mol Biol (2014) 50(3):471–82. doi: 10.1165/rcmb.2013-0348TR PMC4068939 24111585

[27] Eisner MD Anthonisen N Coultas D Kuenzli N Perez-Padilla R Postma D. An Official American Thoracic Society Public Policy Statement: Novel Risk Factors and the Global Burden of Chronic Obstructive Pulmonary Disease. Am J Respir Crit Care Med (2010) 182(5):693–718. doi: 10.1164/rccm.200811-1757ST 20802169

[28] Christenson SA van den Berge M Faiz A Inkamp K Bhakta N Bonser LR. An Airway Epithelial IL-17A Response Signature Identifies a Steroid-Unresponsive COPD Patient Subgroup. J Clin Invest (2019) 129(1):169–81. doi: 10.1172/jci121087 PMC6307967 30383540

[29] Decramer M Janssens W Miravitlles M. Chronic Obstructive Pulmonary Disease. Lancet (2012) 379(9823):1341–51. doi: 10.1016/s0140-6736(11)60968-9 PMC7172377 22314182

[30] Walter RE Wilk JB Larson MG Vasan RS Keaney JF Jr. Lipinska I. Systemic Inflammation and COPD: The Framingham Heart Study. Chest (2008) 133(1):19–25. doi: 10.1378/chest.07-0058 17908709

[31] Manevski M Muthumalage T Devadoss D Sundar IK Wang Q Singh KP. Cellular Stress Responses and Dysfunctional Mitochondrial-Cellular Senescence, and Therapeutics in Chronic Respiratory Diseases. Redox Biol (2020) 33:101443. doi: 10.1016/j.redox.2020.101443 32037306 PMC7251248

[32] Fanucchi S Fok ET Dalla E Shibayama Y Borner K Chang EY. Immune Genes Are Primed for Robust Transcription by Proximal Long Noncoding RNAs Located in Nuclear Compartments. Nat Genet (2019) 51(1):138–50. doi: 10.1038/s41588-018-0298-2 30531872

[33] Wang H Chen L Li D Zeng N Wu Y Wang T. Microarray Analysis of Lung Long Non-Coding RNAs in Cigarette Smoke-Exposed Mouse Model. Oncotarget (2017) 8(70):115647–56. doi: 10.18632/oncotarget.23362 PMC5777800 29383188

[34] Rao W Wang S Duleba M Niroula S Goller K Xie J. Regenerative Metaplastic Clones in COPD Lung Drive Inflammation and Fibrosis. Cell (2020) 181(4):848–864.e18. doi: 10.1016/j.cell.2020.03.047 32298651 PMC7294989

[35] Gindele JA Kiechle T Benediktus K Birk G Brendel M Heinemann F. Intermittent Exposure to Whole Cigarette Smoke Alters the Differentiation of Primary Small Airway Epithelial Cells in the Air-Liquid Interface Culture. Sci Rep (2020) 10(1):6257. doi: 10.1038/s41598-020-63345-5 32277131 PMC7148343

[36] Bodas M Moore AR Subramaniyan B Georgescu C Wren JD Freeman WM. Cigarette Smoke Activates NOTCH3 to Promote Goblet Cell Differentiation in Human Airway Epithelial Cells. Am J Respir Cell Mol Biol (2021) 64(4):426–40. doi: 10.1165/rcmb.2020-0302OC PMC8008804 33444514

[37] Shaykhiev R. Emerging Biology of Persistent Mucous Cell Hyperplasia in COPD. Thorax (2019) 74(1):4–6. doi: 10.1136/thoraxjnl-2018-212271 30266881 PMC6347109

[38] Gu W Yuan Y Wang L Yang H Li S Tang Z. Long non-Coding RNA TUG1 Promotes Airway Remodelling by Suppressing the miR-145-5p/DUSP6 Axis in Cigarette Smoke-Induced COPD. J Cell Mol Med (2019) 23(11):7200–9. doi: 10.1111/jcmm.14389 PMC6815828 31557398

[39] Mei J Zhang Y Lu S Wang J. Long Non-Coding RNA NNT-AS1 Regulates Proliferation, Apoptosis, Inflammation and Airway Remodeling of Chronic Obstructive Pulmonary Disease *via* Targeting miR-582-5p/FBXO11 Axis. BioMed Pharmacother (2020) 129:110326. doi: 10.1016/j.biopha.2020.110326 32768929

[40] Yi E Zhang J Zheng M Zhang Y Liang C Hao B. Long Noncoding RNA IL6-AS1 Is Highly Expressed in Chronic Obstructive Pulmonary Disease and is Associated With Interleukin 6 by Targeting miR-149-5p and Early B-Cell Factor 1. Clin Transl Med (2021) 11(7):e479. doi: 10.1002/ctm2.479 34323408 PMC8288003

[41] Statello L Guo CJ Chen LL Huarte M. Gene Regulation by Long Non-Coding RNAs and Its Biological Functions. Nat Rev Mol Cell Biol (2021) 22(2):96–118. doi: 10.1038/s41580-020-00315-9 33353982 PMC7754182

[42] Caobi A Dutta RK Garbinski LD Esteban-Lopez M Ceyhan Y Andre M. The Impact of CRISPR-Cas9 on Age-Related Disorders: From Pathology to Therapy. Aging Dis (2020) 11(4):895–915. doi: 10.14336/ad.2019.0927 32765953 PMC7390517

[43] Manevski M Devadoss D Castro R Delatorre L Yndart A Jayant RD. Development and Challenges of Nanotherapeutic Formulations for Targeting Mitochondrial Cell Death Pathways in Lung and Brain Degenerative Diseases. Crit Rev BioMed Eng (2020) 48(3):137–52. doi: 10.1615/CritRevBiomedEng.2020034546 33389892

[44] Rogers DF Barnes PJ. Treatment of Airway Mucus Hypersecretion. Ann Med (2006) 38(2):116–25. doi: 10.1080/07853890600585795 16581697

[45] Cerveri I Brusasco V. Revisited Role for Mucus Hypersecretion in the Pathogenesis of COPD. Eur Respir Rev (2010) 19(116):109–12. doi: 10.1183/09059180.00002710 PMC9682580 20956178

[46] Winkle M El-Daly SM Fabbri M Calin GA. Noncoding RNA Therapeutics - Challenges and Potential Solutions. Nat Rev Drug Discov (2021) 20(8):629–51. doi: 10.1038/s41573-021-00219-z PMC8212082 34145432

