# Corrigendum: Increased expression of *LASI* LncRNA regulates the cigarette smoke and COPD associated airway inflammation and mucous cell hyperplasia

**DOI:** 10.3389/fimmu.2022.988069

**Published:** 2022-08-08

**Authors:** Marko Manevski, Dinesh Devadoss, Christopher Long, Shashi P. Singh, Mohd Wasim Nasser, Glen M. Borchert, Madhavan N. Nair, Irfan Rahman, Mohan Sopori, Hitendra S. Chand

**Affiliations:** ^1^ Department of Immunology and Nano-Medicine, Herbert Wertheim College of Medicine, Florida International University, Miami, FL, United States; ^2^ Respiratory Immunology Program, Lovelace Respiratory Research Institute, Albuquerque, NM, United States; ^3^ Department of Biochemistry and Molecular Biology, University of Nebraska Medical Center, Omaha, NE, United States; ^4^ Department of Pharmacology, University of South Alabama, Mobile, AL, United States; ^5^ Department of Environmental Medicine, University of Rochester Medical Center, Rochester, NY, United States

**Keywords:** bronchial epithelial cells, cigarette smoke (CS), chronic obstructive pulmonary disease (COPD), long noncoding RNA (lncRNA), mucus hyperexpression, lncRNA antisense to ICAM-1 (LASI)

In the published article, there was an error in **Table 1** as published. The information about the smoking history (Stop Smoking) for the subjects with ‘No COPD’ was duplicated. The corrected **Table 1** and its caption appear below.

**Table 1:** Demographics of the study cohort of COPD patients with clinically-defined GOLD stage severity and self-reported smoking history.

	No COPD	Mild COPD	Severe COPD
Age*	54.2 ± 3.2	69.2 ± 3.9	65.1 ± 2.7
Gender, M/F	3M/3F	4M/2F	3M/5F
Smoking in PY*	54.0 ± 12.6 (2)	29.9 ± 10.7 (3)	35.0 ± 2.8 (3)
Stop Smoking (Y)*	6.6 ± 1.6	21.4 ± 12.8	11.9 ± 2.5

*Mean, ± SEM; M, Male; F, Female; PY, Packs per Year; Y, Years.

The authors state that this does not change the scientific conclusions of the article in any way. The original article has been updated.

## Publisher’s note

All claims expressed in this article are solely those of the authors and do not necessarily represent those of their affiliated organizations, or those of the publisher, the editors and the reviewers. Any product that may be evaluated in this article, or claim that may be made by its manufacturer, is not guaranteed or endorsed by the publisher.

