# Supplementary material for: Increased Expression of LASI lncRNA Regulates the Cigarette Smoke and COPD Associated Airway Inflammation and Mucous Cell Hyperplasia
Source: Front Immunol. 2022 Jun 14;13:803362. doi: 10.3389/fimmu.2022.803362 (PMC9237255; doi:10.3389/fimmu.2022.803362)
Supplement: Supplementary file 1 [file DataSheet_1.docx]

**Online Supplemental Data**

Increased Expression of *LASI* LncRNA Regulates the Cigarette Smoke and COPD Associated Airway Inflammation and Mucous Cell Hyperplasia

Marko Manevski^1^, Dinesh Devadoss^1^, Christopher Long^1^, Shashi Singh^2^, Mohd Wasim Nasser^3^, Glen M. Borchert^4^, Madhavan Nair^1^, Irfan Rahman^5^, Mohan Sopori^2^, and Hitendra S. Chand^1^

^1^Department of Immunology and Nano-Medicine, Herbert Wertheim College of Medicine, Florida International University, Miami, FL, USA; ^2^Lovelace Respiratory Research Institute, Albuquerque, NM, USA; ^3^Department of Biochemistry and Molecular Biology, University of Nebraska Medical Center, Omaha, NE, USA; ^4^Department of Pharmacology, University of South Alabama, Mobile, AL, USA; ^5^Department of Environmental Medicine, University of Rochester Medical Center, Rochester, NY, USA

***Corresponding Author:** Hitendra S. Chand, PhD

Department of Immunology and Nano-Medicine,

Herbert Wertheim College of Medicine,

Florida International University,

Miami, FL 33199

Tel: (305) 348-1472

E-mail: [hchand@fiu.edu](mailto:hchand@fiu.edu)


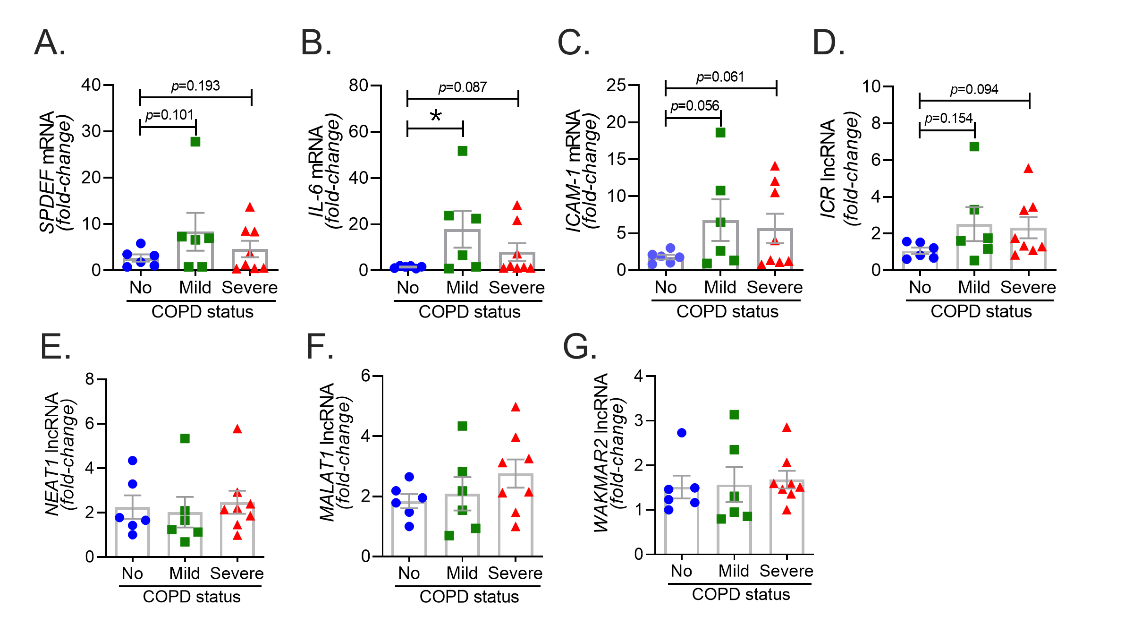


**Supplemental Figure S1. Expression levels of select inflammatory factors and lncRNAs in the lung tissue samples of mild and severe COPD donors versus the controls with no COPD.** Relative quantities of *SPDEF* **(A.)***, IL-6* **(B.)**, and *ICAM-1* **(C.)** mRNA levels in lung tissues from mild and severe COPD subjects compared to control subjects with no COPD, analyzed by qRT-PCR. Relative quantities of lncRNA levels of *ICR* **(D.)**, *NEAT1* **(E.)***, MALAT1* **(F.)**, and *WAKMAR2* **(G.)**. Data shown as mean±SEM (n=6-8/gp); data analysed by ANOVA, **p*<0.05.


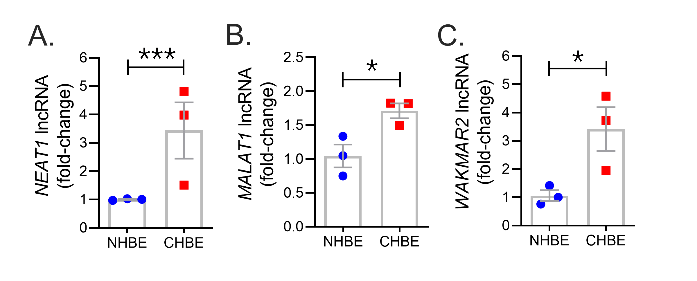


**Supplemental Figure S2. Expression levels of select lncRNAs in differentiated NHBEs and CHBEs cultured on air-liquid interface.** Relative quantity of lncRNA levels of *NEAT1* **(A.)***,* *MALAT1* **(B.)**, and *WAKMAR2* **(C.)** in unstimulated NHBEs and CHBEs. Data shown as mean±SEM (n=3/gp); data analyzed by student’s t-test, **p*<0.05; ****p*<0.001.


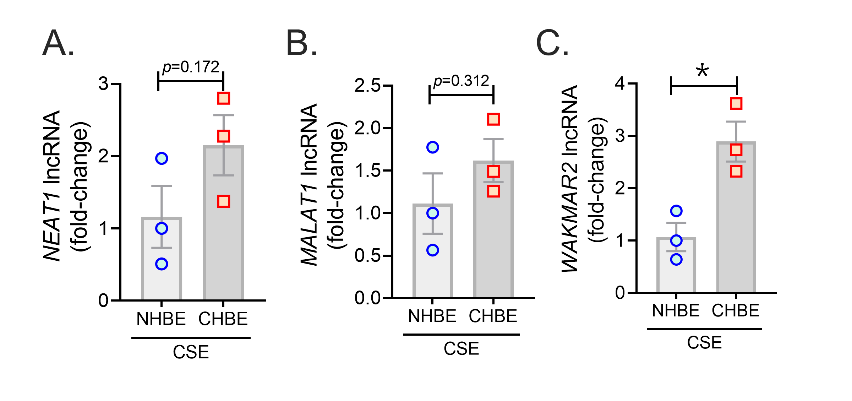


**Supplemental Figure S3. Expression levels of select lncRNAs in CSE-treated NHBEs and CHBEs.** Relative quantity of lncRNA levels of *NEAT1* **(A.)***,* *MALAT1* **(B.)**, and *WAKMAR2* **(C.)** in CSE-treated NHBEs and CHBEs. Data shown as mean±SEM (n=3/gp); data analyzed by student’s t-test, **p*<0.05; ****p*<0.001.


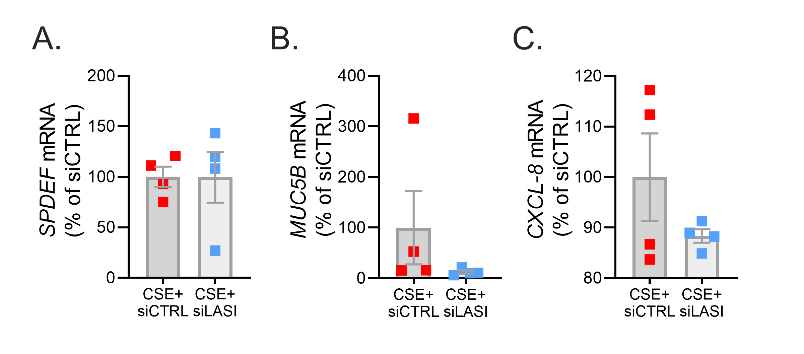


**Supplemental Figure S4. Effect of RNA silencing-mediated knockdown of *LASI* lncRNA on *SPDEF*, *MUC5B,* and *CXCL-8* mRNA expression in CSE-treated CHBEs.** Relative expression levels of *SPDEF* **(A.)***,* *MUC5B* **(B.)***,* and *CXCL-8* **(C.)** mRNAs as determined by qRT-PCR. Data shown as mean ± SEM compared to CSE+siCTRL cells; n=4/gp; data analyzed by student’s t-test.


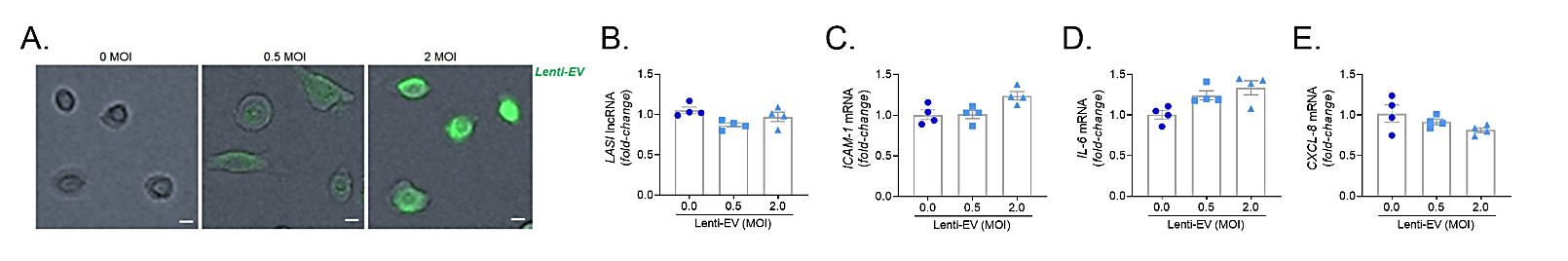


**Supplemental Figure S5. Control empty-vector lentiviral preparation transduction do not alter the expression of airway inflammatory factors. (A.)** Representative micrographs of cells transduced with 0, 0.5, and 2 MOI of empty vector lentiviral preparation (Lenti-EV) with GFP reporter (shown in green), scale – 5µm. Cells harvested forty-eight hours post-transduction with Lenti-EV were analyzed for expression levels of *LASI* lncRNA **(B.)**, and mRNA levels of *ICAM-1* **(C.)***,* *IL-6* **(D.)**, and *CXCL-8* **(E.)** by qRT-PCR. Data shown as mean±SEM compared to mock-transduced cells (0 MOI); data analyzed by ANOVA with multiple comparisons.
